# PlAtg8-mediated autophagy regulates vegetative growth, sporangial cleavage, and pathogenesis in *Peronophythora litchii*

**DOI:** 10.1128/spectrum.03531-23

**Published:** 2023-12-12

**Authors:** Ge Yu, Wenqiang Li, Chengdong Yang, Xue Zhang, Manfei Luo, Taixu Chen, Xuejian Wang, Rongbo Wang, Qinghe Chen

**Affiliations:** 1 School of Tropical Agriculture and Forestry, School of Breeding and Multiplication (Sanya Institute of Breeding and Multiplication), Hainan University, Sanya, China; 2 Key Laboratory of Green Prevention and Control of Tropical Plant Diseases and Pests, Ministry of Education, Hainan University, Haikou, China; 3 Fujian Key Laboratory for Monitoring and Integrated Management of Crop Pests, Institute of Plant Protection, Fujian Academy of Agricultural Sciences, Fuzhou, China; Chinese Academy of Sciences, Shanghai, China

**Keywords:** PlAtg8, sporangial cleavage, autophagosome formation, pathogenicity, *Peronophythora litchii*

## Abstract

Autophagy is a conserved cellular metabolic process in eukaryotes that transports damaged or redundant intracellular organelles and proteins to lysosomes or vacuoles for degradation and recycling. Atg8 is not only essential for autophagosome formation but also required for fungal development and pathogenicity, but its function is unknown in *Peronophythora litchii*. In this study, PlAtg8, a yeast Atg8 homolog, is deleted by the CRISPR/Cas9 system coupled with PEG-mediated protoplast transformation in *P. litchii*. Compared with the wild-type strain, the absence of PlAtg8 affects autophagosome formation. The colony growth rate, number of sporangia, and zoospore release rate are significantly decreased, and the oospores are undetected in Δ*Platg8*. The pathogenicity test shows that the lesion areas on litchi leaves and fruits caused by Δ*Platg8* are significantly attenuated compared with the wild type, indicating that PlAtg8 is also required for the pathogenicity of *P. litchii*. Moreover, the cleavage rate of sporangia in Δ*Platg8* is significantly decreased compared to the wild type by FM4-64 staining, which is probably the main reason for the lower zoospores release rate in Δ*Platg8*. These results suggest that PlAtg8 not only regulates autophagy but also participates in the growth and development of *P. litchii*.

**IMPORTANCE:**

*Peronophythora litchii* is the pathogen of litchi downy blight, which is the most serious disease in litchi. Autophagy is an evolutionarily conserved catabolic process in eukaryotes. Atg8 is a core protein of the autophagic pathway, which modulates growth and pathogenicity in the oomycete *P. litchii*. In *P. litchii*, CRISPR/Cas9-mediated knockout of the *PlATG8* impaired autophagosome formation. *PlATG8* knockout mutants exhibited attenuated colony expansion, sporangia production, zoospore discharge, and virulence on litchi leaves and fruits. The reduction in zoospore release was likely underpinned by impaired sporangial cleavage. Thus, in addition to governing autophagic flux, PlAtg8 is indispensable for vegetative growth and infection of *P. litchii*.

## INTRODUCTION

Oomycetes, although morphologically similar to fungi, differ from fungi in terms of their growth, development, and pathogenic mechanisms, making them significant plant pathogens (1). To date, over 1,200 species of oomycetes have been identified, causing serious damage to forestry, agriculture, and ecosystems, and tens of billions of dollars in economic losses each year (2 – 4). For example, potatoes, soybeans, and cucurbits are severely damaged by *Phytophthora infestans*, *P. sojae*, and *P. capsici. Peronophythora litchii* is also an oomycete, and it is one of the most important diseases in litchi production (5). However, the molecular mechanisms underlying the growth, development, and pathogenesis of *P. litchii* are largely unknown.

Autophagy is a highly conserved intracellular degradation process in eukaryotic cells that plays a crucial role in maintaining cellular homeostasis (6). During this process, damaged or surplus proteins and organelles are transported to vacuoles (in yeast, fungi, and plant cells) or lysosomes (in animal cells) for degradation, thereby participating in new nutrient cycles (7, 8). In recent decades, increasing evidence has shown that autophagy is critical for the growth and infection-related development of plant pathogenic fungi. For example, deletion of any of the 16 *ATG* genes required for the core autophagy process in *Magnaporthe oryzae* affects its virulence (9). In *Fusarium graminearum*, deletion of any autophagy gene except for *FgATG17* would result in loss of virulence (10). In *P. litchii*, PlAtg6a plays an important role in autophagy, growth, sexual, and asexual reproduction (11).

Atg8 is a ubiquitin-like protein that is the core protein of the autophagic pathway. During autophagy, Atg8 is initially cleaved at the C-terminus by Atg4 to expose glycine residues (12) and then ubiquitinated by Atg7 and Atg3. Finally, in the presence of the Atg12-Atg5-Atg16 complex, the glycine residue binds to the amine moiety of phosphatidylethanolamine (PE) to form the Atg8-PE complex, which is involved in autophagosome formation (13). In yeast and other fungi, deletion of *ATG8* results in the inability to form autophagosomes and hinders the normal function of autophagy (6, 14). Atg8 is also involved in the growth, development, and pathogenicity of plant pathogens. In *Botrytis cinerea*, BcAtg8 plays an important role in the regulation of autophagy in the development, pathogenesis, and lipid metabolism (15). FvAtg4 and FvAtg8 contribute to pathogenicity by regulating the autophagic pathway to control lipid function and toxin synthesis in *Fusarium verticillioides* during invasion (16). In *Ustilaginoidea virens*, the absence of UvAtg8 affects nutrient growth, conidial number, and ability to cope with stress, and significantly reduces the virulence (17).

However, the mechanisms by which PlAtg8 regulates the growth and development, sexual and asexual reproduction, and pathogenicity of *P. litchii* are unknown. In this study, we identified a ScAtg8 homolog, PlAtg8, in *P. litchii* and investigated how PlAtg8 influences the mycelial growth, asexual and sexual reproduction, pathogenicity, and autophagy of *P. litchii*.

## MATERIALS AND METHODS

### Sequence analysis

The genome and protein sequences of *P. litchii* were obtained from Nanjing Agricultural University, and other sequences were downloaded from FungiDB (https://fungidb.org/fungidb/app/). The protein sequences were aligned by CLUSTALW (https://www.genome.jp/tools-bin/clustalw) and adjusted by ESPript3.0 (https://espript.ibcp.fr/ESPript/cgi-bin/ESPript.cgi). MEGA 11 with a neighbor-joining algorithm was used for phylogenetic analysis.

### Strains and culture conditions

The wild-type strain (WT) of *P. litchii*, SHS3, was provided by Nanjing Agricultural University and used for transformation experiments in this study. WT was transformed with the empty vector to produce the control strain referred to as EV. All strains were cultured on V8 agar medium (100 mL V8 vegetable juice, 20 g agar, and 1 L water) at 25°C. The minimal medium contained 0.5 g KCl, 0.8 g KH_2_PO_4_, 1 g K_2_HPO_4_, 0.5 g MgSO_4_ **·**7H_2_O, 1 mL trace elements solution, and 1 L water, pH 6.5. Trace elements solution contained FeSO_4_ **·**7H_2_O 5 g, ZnSO_4_ **·**7H_2_O 22 g, H_3_BO_3_ 11 g, MnCl_2_ **·**4H_2_O 5 g, CoCl_2_ **·**6H_2_O 1.6 g, CuSO_4_ **·**5H_2_O 1.6 g, (NH_4_)_6_Mo_7_O_24_ **·**4H_2_O 1.1 g, ethylenediaminetetraacetic acid 50 g, and 1 L water (18). A minimal medium without N source (MM-N) was used for autophagy induction.

### Construction of *PlATG8* deletion and complementation mutants

To generate a deletion mutant of *PlATG8* in *P. litchii*, the CRISPR/Cas9 system and PEG-mediated protoplast transformation were used according to the previously described method (19). For the deletion experiment, the 1 kb of homologous flanking sequences on the upstream and downstream side, respectively, was amplified from WT using primers *PlATG8* UP F/R and *PlATG8* DN F/R. The HPH cassette was amplified from the pCX62 vector using primers *HPH* F/R. The three amplicons were inserted into the pBluescript SK II^+^ (pBS-SK II^+^) vector. Two sgRNAs targeting *PlATG8* were designed using EuPaGDT (http://grna.ctegd.uga.edu/) and cloned into the pYF515 vector. The two constructed vectors were co-transformed into protoplasts of WT. For the complementation experiment, the ORF sequence of *PlATG8* was amplified from WT and inserted into the pBS-SK II^+^ vector with upstream and downstream homologous flanking sequences. The pBS-SK II^+^ and pYF515 were co-transformed into protoplasts of Δ*Platg8*. Primers used in this study are shown in Table S1.

### qRT-PCR analysis

Total RNA was extracted from mycelium, sporangia, zoospores, cysts, and germinated cysts using the Eastep Super Total RNA Extraction Kit (Promega, China). ToloScript RT EasyMix for qPCR (with 2-Step gDNA Erase-Out) (Tolobio, China) was used for first-strand cDNA synthesis. The expression of *PlATG8* was analyzed using 2 × Q3 SYBR qPCR Master mix (Tolobio, China) and calculated using the 2^−ΔΔCT^ method (20). *ACTIN* gene in *P. litchii* was used as a control (21). Primers used in this study are shown in Table S1.

### Growth and development

To test colony diameter, all strains were cultured on V8 agar medium at 25°C and the colony diameter was measured after 5 days. To calculate the number of sporangia, the sporangia were collected from the colony cultured on V8 agar medium at 25°C after 5 days with 5 mL double-sterilized water to obtain the sporangia suspension. For zoospore release, the sporangia were collected with 1.5 mL double-sterilized water to obtain the sporangia suspension, which was induced at 12°C for 0.5 h and 2 h.

To calculate the number of oospores, mycelial plugs of 5 mm diameter were obtained randomly from a 10-day-old colony cultured on V8 agar medium at 25°C. Assays above all were repeated three times. The significant differences were analyzed with one-way ANOVA.

### Generation of the GFP-PlAtg8 strain

To express *GFP-PlATG8* in *P. litchii*, the *GFP* sequence was amplified from the pKNT vector, and the *PlATG8* sequence was amplified from WT. The two amplicons were inserted into the pKNT vector, which was transformed into protoplasts of WT. The transformants were verified by PCR and fluorescence microscope.

### Staining and microscopy

For FM4-64 staining, the sporangia were collected from the colony cultured on V8 agar medium at 25°C after 5 days and induced at 12°C for 0.5 h and 2 h. The sporangia suspension was incubated with 1 µg/mL FM4-64 for 5 min at room temperature. For DAPI (4′,6-diamidino-2-phenylindole) staining, the sporangia suspension was incubated with DAPI for 5 min at room temperature. After staining, samples were observed with a fluorescence microscope.

### Pathogenicity assays

The pathogenicity of *P. litchii* was tested on litchi leaves and fruits using mycelia plugs with 5 mm diameter obtained from the edge of a 5-day-old colony cultured on V8 agar medium. For the pathogenicity test on leaves, the inoculated leaves were cultured at 25°C, and the lesion area was measured at 36 hpi (hours post-inoculation). For the pathogenicity test on leaves, the lesion area was measured at 48 hpi. The V8 agar media without mycelia were used as a mock. The lesion area was calculated by ImageJ, and significant differences were analyzed with one-way ANOVA. Assays above all were repeated three times.

### The activity of laccase and extracellular oxidase

To investigate the activity of laccase, the mycelial plugs of WT, EV, Δ*Platg8*, and Δ*Platg8*-C13 were inoculated on V8 medium with ABTS (2,2′-azinobis(3-ethylbenzothiazoline-6-sulfonic acid)) and incubated at 25°C in the dark for 15 days.

To investigate the activity of extracellular oxidase, the mycelial plugs of WT, EV, Δ*Platg8*, and Δ*Platg8*-C13 were inoculated on Plich medium with Congo red (CR) and incubated at 25°C in the dark. The transparent circle was measured at 24 hpi.

### Sensitivity to various stress

To investigate the sensitivity of Δ*Platg8* mutants to various stresses, mycelial plugs of WT, EV, Δ*Platg8*, and Δ*Platg8*-C13 were inoculated on V8 medium and incubated at 25°C in the dark for 5 days. Different concentrations of sodium dodecyl sulfate (SDS), glycerol, CR, NaCl, KCl, and H_2_O_2_ were added to V8 medium. The growth inhibition rate was calculated as follows: Growth inhibition rate (%) = (Growth diameter on stress-free plates − Growth diameter on stress plates)/Growth diameter on stress-free plates × 100%.

## RESULTS

### Sequence analysis of PlAtg8 in *P. litchii*

To identify the Atg8 homolog in *P. litchii*, the Atg8 protein sequence of *S. cerevisiae* was employed as a query for a BLASTP search in the *P. litchii* protein database, hereafter referred to as PlAtg8. We searched for the orthologs of PlAtg8 in oomycete and fungus species, including *P. capsici*, *P. infestans*, *P. parasitica*, *P. sojae*, *M. oryzae,* and *F. graminearum*. All these species contained orthologs of PlAtg8, suggesting that PlAtg8 orthologs are ubiquitous in oomycetes and fungi (Fig. S1A). Phylogenetic analysis of these species showed that PlAtg8 was most similar to PcAtg8 (Fig. S1B). These results indicate that Atg8 is closely conserved among different species and that PlAtg8 may have essential functions in *P. litchii*.

To investigate the biological function of PlAtg8 in *P. litchii*, the expression levels of the *PlATG8* gene were analyzed using real-time quantitative fluorescence PCR. As shown in Fig. S1C, the expression levels of the *PlATG8* gene were upregulated in zoospores (ZO), cysts (CY), and cysts germination (CG). These results suggest that *PlATG8* is involved in the asexual development of *P. litchii*.

### Knock out and complementation of *PlATG8*

Using the CRISPR/Cas9 system and PEG-mediated protoplast transformation, we generated a deletion mutant of *PlATG8* in *P. litchii* to study the function of *PlATG8*. Two sgRNAs were designed to target the *PlATG8* coding region. Candidate transformants obtained through G418 selection were further validated as mutants by genomic PCR and sequencing techniques. In the end, we obtained a *PlATG8* knockout mutant from 51 transformants (Fig. S2B). To confirm that the disruption of *PlATG8* was responsible for the phenotype described below, an *in situ* complementation assay was conducted to generate the *PlATG8* complemented mutants. Moreover, an EV control strain was generated in *P. litchii* through transformation with empty vectors (Fig. S2B). Furthermore, qRT-PCR analysis demonstrated an absence of *PlATG8* expression in the mutant strain (Fig. S2C).

### PlAtg8 is required for autophagy

**Fig 1:**
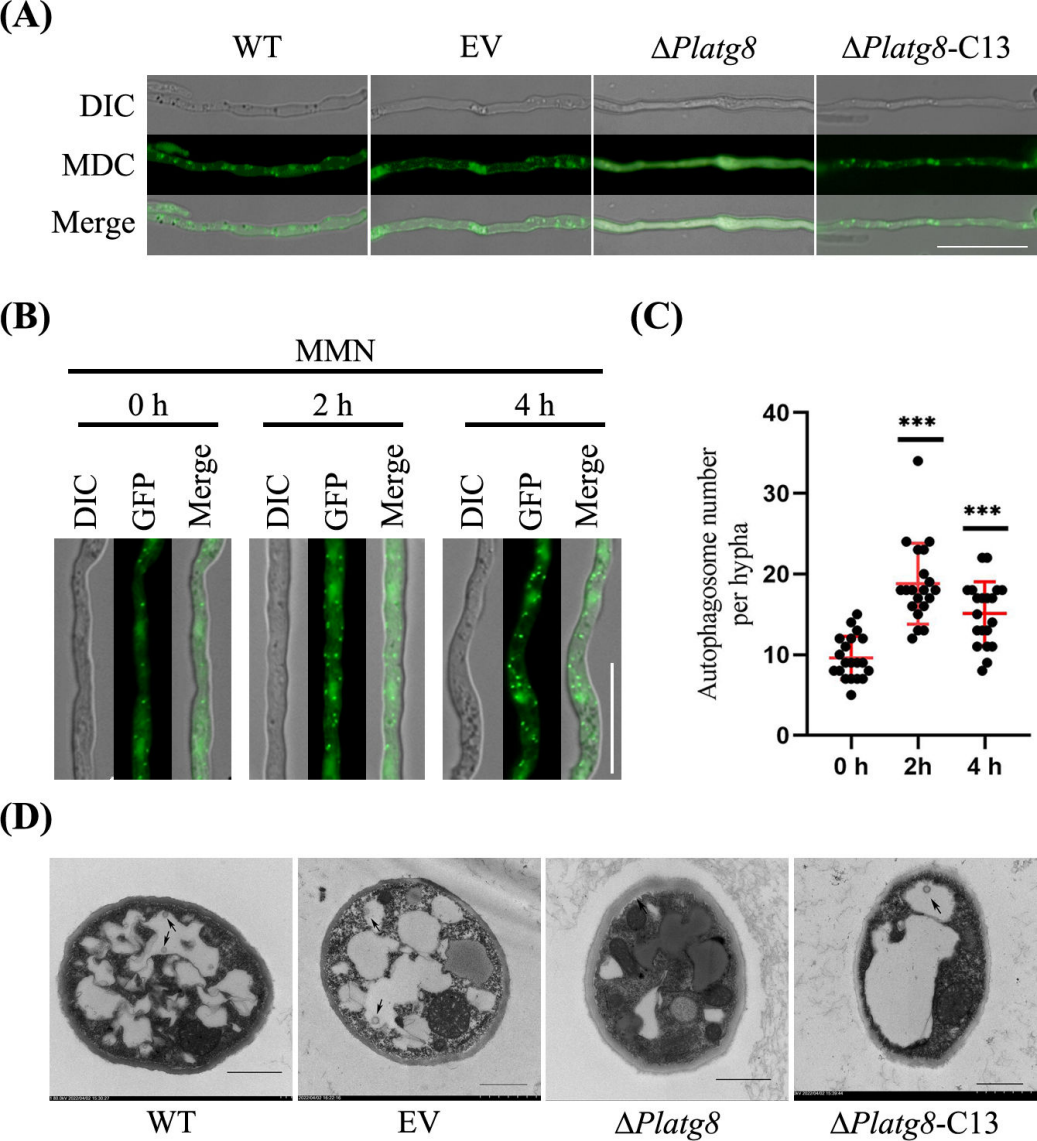
PlAtg8 is required for autophagy in *P. litchii*. (**A**) Mycelia from WT, EV, Δ*Platg8*, and Δ*Platg8*-C13 strains under nitrogen starvation were stained with MDC and visualized by fluorescence microscopy. Scale bar = 20 µm. (**B**) Microscopic observation of GFP-PlAtg8 strain under nitrogen-rich and starvation conditions. The strain was grown in V8 medium for 3 days, then transferred to MM-N medium for 2 h and 4 h. Scale bar = 20 µm. (**C**) Quantification of autophagosome numbers. Data were analyzed by one-way ANOVA. Asterisks denote significant differences (***P < 0.0001). (**D**) Transmission electron micrographs (TEM) of nitrogen-starved mycelia from WT, EV, Δ*Platg8*, and Δ*Platg8*-C13. The black arrow indicates autophagosomes.

As a highly conserved autophagy protein in eukaryotes, Atg8 is often employed as a molecular marker to study autophagic mechanisms (22). To explore the function of PlAtg8 in autophagy, monodansylcadaverine (MDC) staining was used to detect autophagosomes. The strains wild-type SHS3, control EV, *PlATG8* knockout mutant (Δ*Platg8*), and complemented strain (Δ*Platg8*-C13) were cultured in V8 medium and shifted into nitrogen starvation medium (MM-N) with 2 mM PMSF (phenylmethylsulfonyl fluoride) for 4 h. As shown in Fig. 1A, a large number of punctate autophagosomes were observed in WT, EV, and Δ*Platg8*-C13, whereas no autophagosomes appeared in Δ*Platg8*. The result showed that the absence of PlAtg8 affects the formation of autophagosomes.

To further investigate the autophagy, the GFP-PlAtg8 was transformed into WT to observe the localization of PlAtg8 in *P. litchii*. The strain expressing GFP-PlAtg8 was cultured in V8 medium and transferred to MM-N medium with 2 mM PMSF for 0 h and 4 h, respectively. The GFP-PlAtg8 signal was predominantly localized to punctate structures (Fig. 1B), which is consistent with the findings from MDC staining. The GFP-PlAtg8 strain exhibited a weak GFP signal and a few autophagosomes when grown on a V8 medium. However, the GFP signal was strong and the number of autophagosomes increased significantly after 4 h of starvation in MM-N (Fig. 1C). The results indicate that GFP-PlAtg8 was primarily found in the cytoplasm and exhibited rapid accumulation in autophagosome during nitrogen starvation.

Moreover, autophagosomes were observed by transmission electron microscopy (TEM). The hyphae of WT, EV, Δ*Platg8*, and Δ*Platg8*-C13 were treated with 100 nmol/L rapamycin for 4 h. Subsequently, autophagosomes (marked by black arrows) were obviously observed in the vacuole of WT and EV using TEM (Fig. 1D). However, only a few autophagosomes were formed in Δ*Platg8*. These results suggest that PlAtg8 is involved in the formation of autophagosomes and is essential for autophagy.

### PlAtg8 is required for the pathogenicity

**Fig 2:**
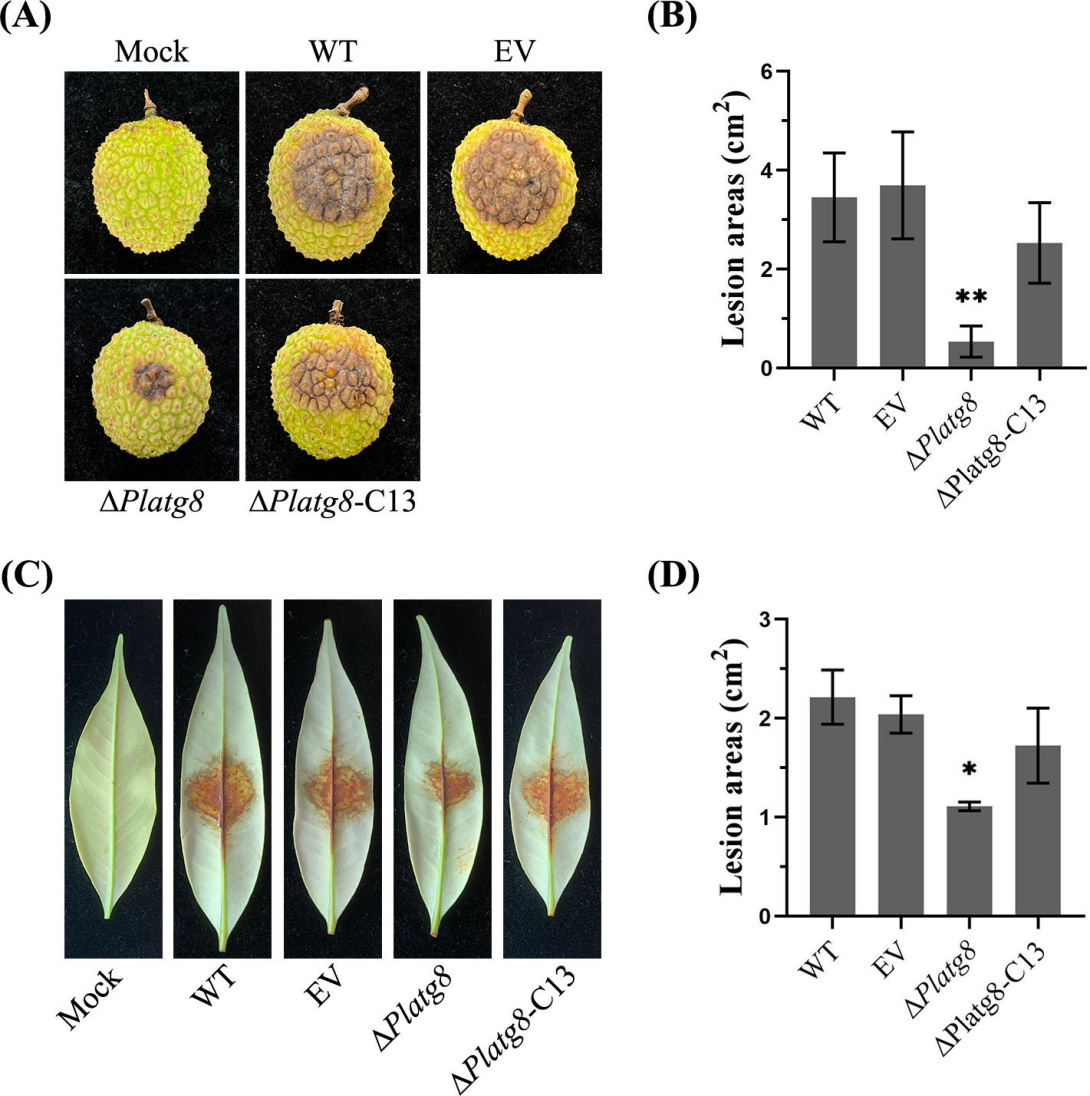
PlAtg8 is required for the pathogenicity. (**A**) Disease symptoms on fruits at 48 hpi with WT, EV, Δ*Platg8*, and Δ*Platg8*-C13 strains. (**B**) The lesion area on litchi fruits was calculated by ImageJ. (**C**) Disease symptoms on leaves at 36 hpi with WT, EV, Δ*Platg8*, and Δ*Platg8*-C13 strains. (**D**) The lesion area on litchi leaves was calculated by ImageJ. Data were analyzed by one-way ANOVA. Asterisks denote significant differences (**P < 0.05). These experiments were repeated three times.

To investigate the role of PlAtg8 in the pathogenicity of *P. litchii*, mycelial plugs of WT, EV, Δ*Platg8*, and Δ*Platg8*-C13 strains were inoculated on litchi leaves and fruits. After 48 h post-inoculation (hpi), the Δ*Platg8* strains exhibited significantly smaller lesions on fruit compared to WT, EV, and Δ*Platg8-*C13 strains (Fig. 2A and B). A similar reduction in pathogenicity was observed for the Δ*Platg8* mutants during leaf infection assays (Fig. 2C and D). These results demonstrate that PlAtg8 is critical for the pathogenicity of *P. litchii*. As sporangia and zoospores are essential for *P. litchii* infection, we hypothesized the loss of PlAtg8 may lead to developmental deficiencies in these structures, consequently attenuating pathogenicity. Subsequent experiments provided evidence to support this hypothesis.

### PlAtg8 is required for the mycelial growth

**Fig 3:**
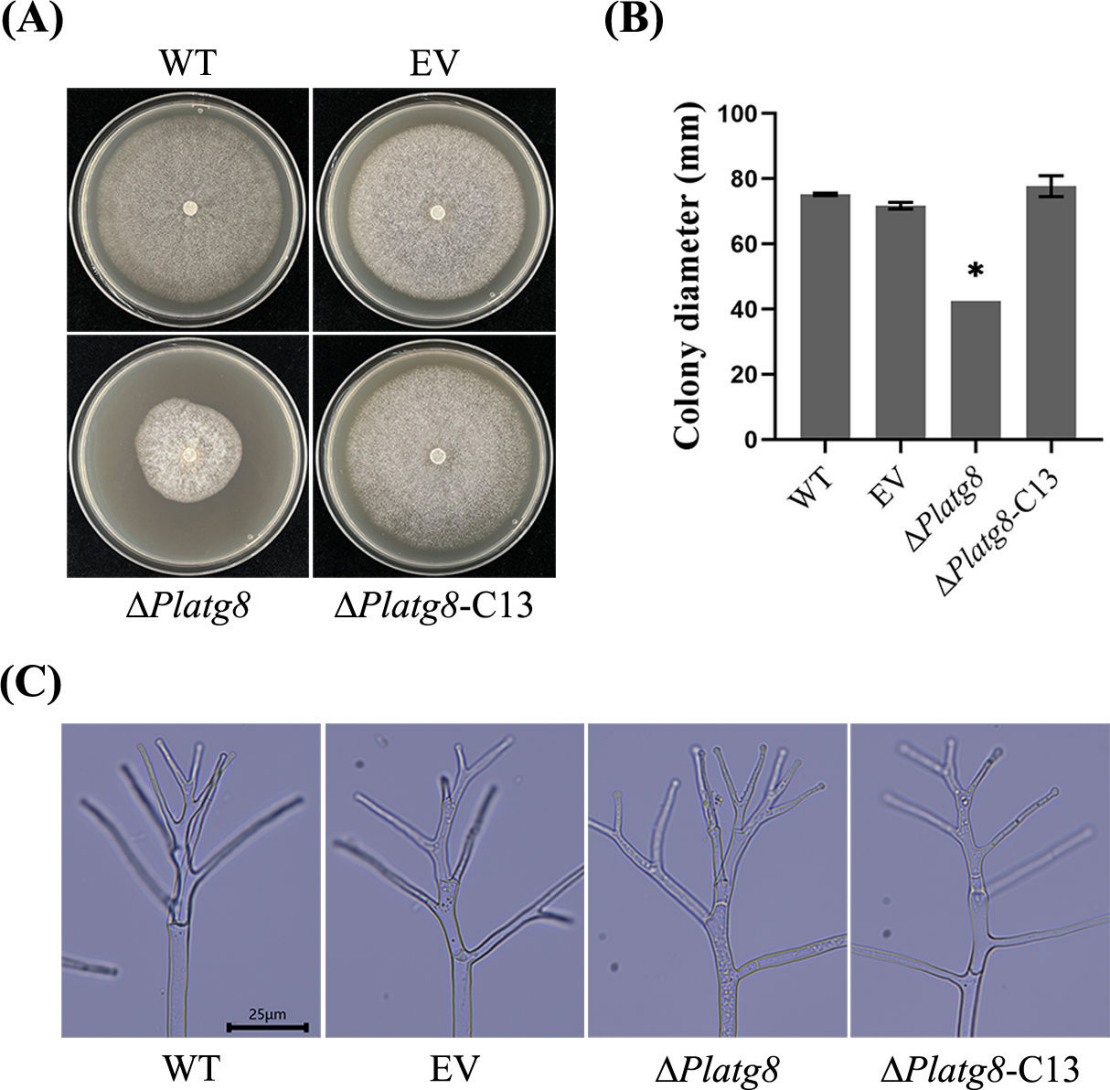
PlAtg8 is required for mycelial growth. (**A**) The WT, EV, Δ*Platg8*, and Δ*Platg8*-C13 strains were grown on V8 medium at 25°C for 5 d. (**B**) Colony diameters of each strain on V8 medium. Data were analyzed by one-way ANOVA. Asterisks denote significant differences (*P < 0.05). These experiments were repeated three times. (**C**) Mycelial morphology of each strain. Scale bar = 25 µm.

To investigate the importance of PlAtg8 in mycelial growth, strains including WT, EV, Δ*Platg8,* and Δ*Platg8*-C13 were cultured on V8 medium. After incubation at 25°C for 5 days, the growth of Δ*Platg8* was significantly slower than that of WT and Δ*Platg8*-C13 (Fig. 3A and B). The aerial mycelium of Δ*Platg8* was more densely compared with that of WT and Δ*Platg8*-C13. Furthermore, microscopic examination showed that the hyphae of Δ*Platg8* exhibited normal morphology comparable to WT (Fig. 3C). These results indicate that PlAtg8 is required for mycelial growth but not for morphogenesis in *P. litchii*.

### PlAtg8 is necessary for the asexual and sexual reproduction

**Fig 4:**
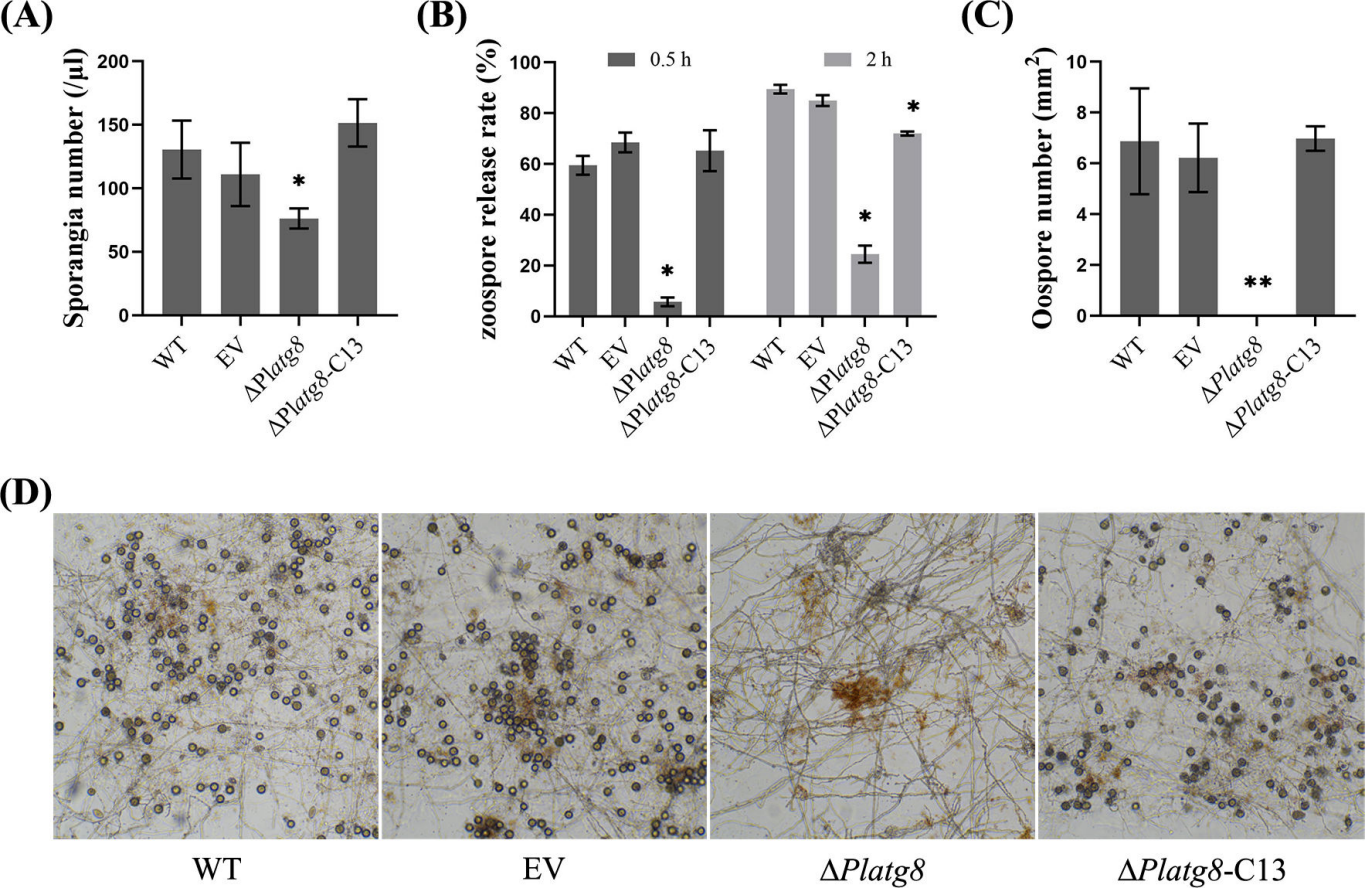
PlAtg8 is required for asexual and sexual reproduction. (**A**) Quantification of sporangia numbers. (**B**) Quantification of zoospore release rate. (**C**) Quantification of oospore numbers. (**D**) Oospore production by WT, EV, Δ*Platg8*, and Δ*Platg8*-C13 strains after 15 days of growth on V8 medium at 25°C. Data were analyzed by One-way ANOVA. Asterisks denote significant differences (*P < 0.05).

Asexual and sexual reproductions are the most important biological processes in the life cycle of oomycetes and are responsible for disease epidemics. In the asexual stage, sporangia are produced and then zoospores are released. The sporangia of WT, EV, Δ*Platg8*, and Δ*Platg8*-C13 were collected and calculated to determine whether *PlATG8* is related to sporangia production and zoospores release rate. The number of sporangia was clearly decreased in Δ*Platg8* compared with WT, EV, and Δ*Platg8*-C13 (Fig. 4A). The results suggest that the disruption of PlAtg8 affects sporangia production. Zoospores are released when sporangia are induced at 12°C in water, and we calculated the zoospore release rates at 0.5 and 2 h. The zoospore release rate of Δ*Platg8* was significantly lower compared to the WT, and even after 2 h of induction, the zoospore release rate of Δ*Platg8* was still less than half that of WT (Fig. 4B). These results suggest that PlAtg8 plays an important role in sporangia production and zoospore release.

Oospores are the main source of primary infection for the next year and are an important part of the disease cycle (23). To investigate the function of *PlATG8* in sexual reproduction, the number of oospores was measured in WT and Δ*Platg8* cultured on carrot agar (CA) medium for 10 days. The results showed that Δ*Platg8* completely lost the ability to produce oospores (Fig. 4C and D). This result suggests that PlAtg8 is required for oospore production by *P. litchii*.

### PlAtg8 is required for sporangial cleavage

**Fig 5:**
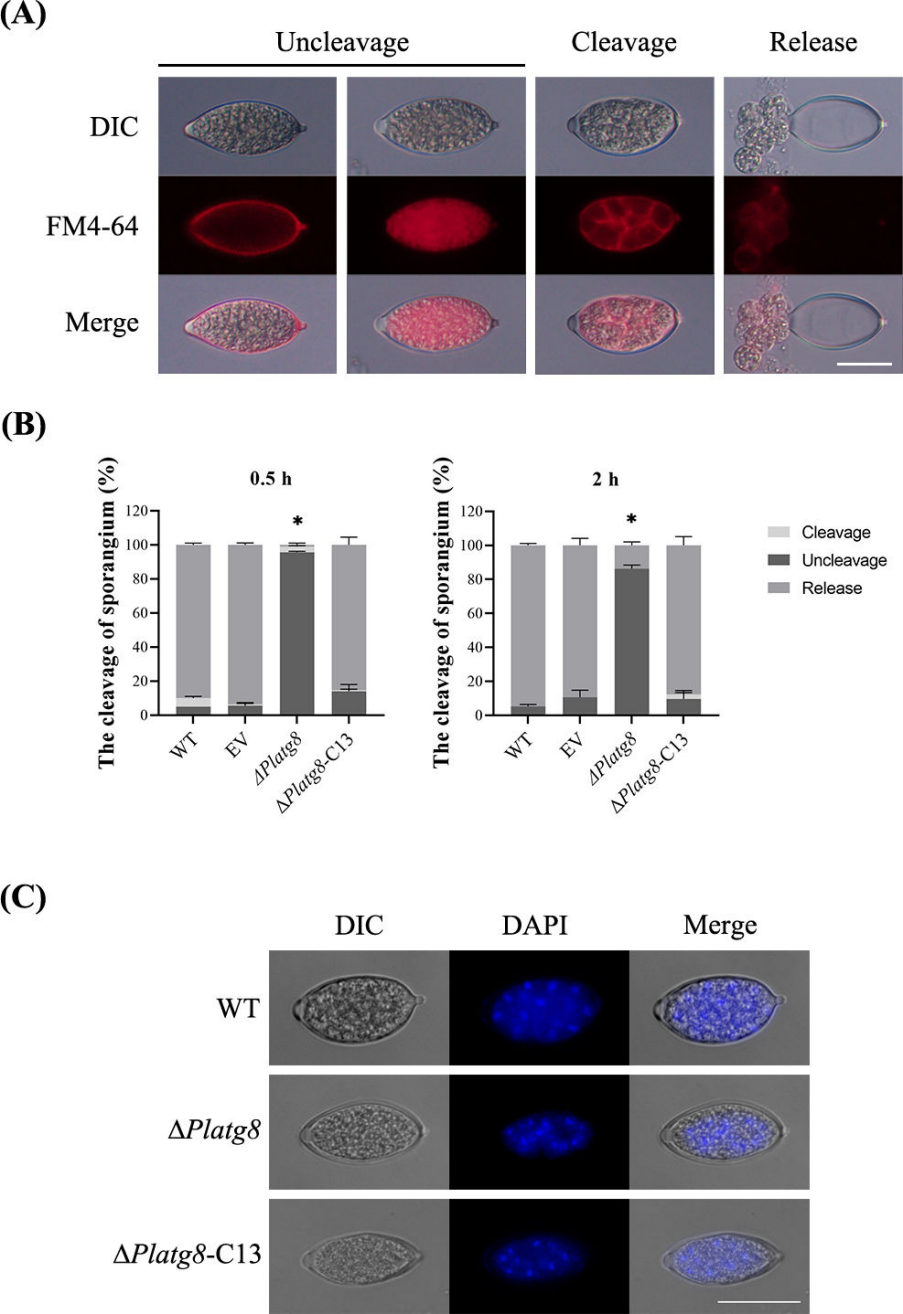
PlAtg8 is required for sporangia cleavage. (**A**) Morphological progression of sporangia during cytoplasmic cleavage. The plasma membrane was stained by FM4-64. (**B**) Quantification of sporangial cleavage rates at 0.5 h and 2 h post-induction. Data were analyzed by one-way ANOVA. Asterisks denote significant differences (*P < 0.05). (**C**) The nuclear location of sporangia during cytoplasmic cleavage. The nuclei were stained by DAPI. Scale bar = 20 µm.

Based on the above experiments, we found that deletion of *PlATG8* impaired the number of sporangia and zoospore release. We evaluated the expression level of *PlATG8* in mycelia (MY), sporangia (SP), zoospore (ZO), cyst (CY), and germination of the cyst (GC), the result showed that *PlATG8* was significantly up-regulated in zoospore (Fig. S1C). Before zoospore release, the cytoplasm of sporangia was cleaved by nucleus-enveloping membrane networks (24). To clarify the underlying reasons for the reduction in the zoospore release rate, the FM4-64 and DAPI were used to observe sporangia cleavage. In the FM4-64 stain assay, consistent with the zoospore release rate, sporangial cleavage was significantly impaired in Δ*Platg8* compared to WT, EV, and Δ*Platg8*-C13 (Fig. 5A and B). After 0.5 h of induction, more than 80% sporangia were cleaved and released in WT, EV, and Δ*Platg8*-C13, and less than 5% sporangia were cleaved and released in Δ*Platg8*. After 2 h of induction, all cleaved sporangia were released in WT, EV, and Δ*Platg8*-C13, and still, less than 20% of sporangia were cleaved and released in Δ*Platg8*. This result suggests that PlAtg8 is required for sporangial cleavage.

To further confirm the result of sporangial cleavage, a DAPI stain assay was used to observe the sporangial nucleus. The nucleus of developed zoospores was spaced regularly in the sporangia of WT. By contrast, the nucleus was massed in the sporangia of Δ*Platg8* (Fig. 5C). These results demonstrated that PlAtg8 is involved in sporangial cleavage during zoospore development.

### PlAtg8 is required for laccase activity but not for extracellular oxidase

**Fig 6:**
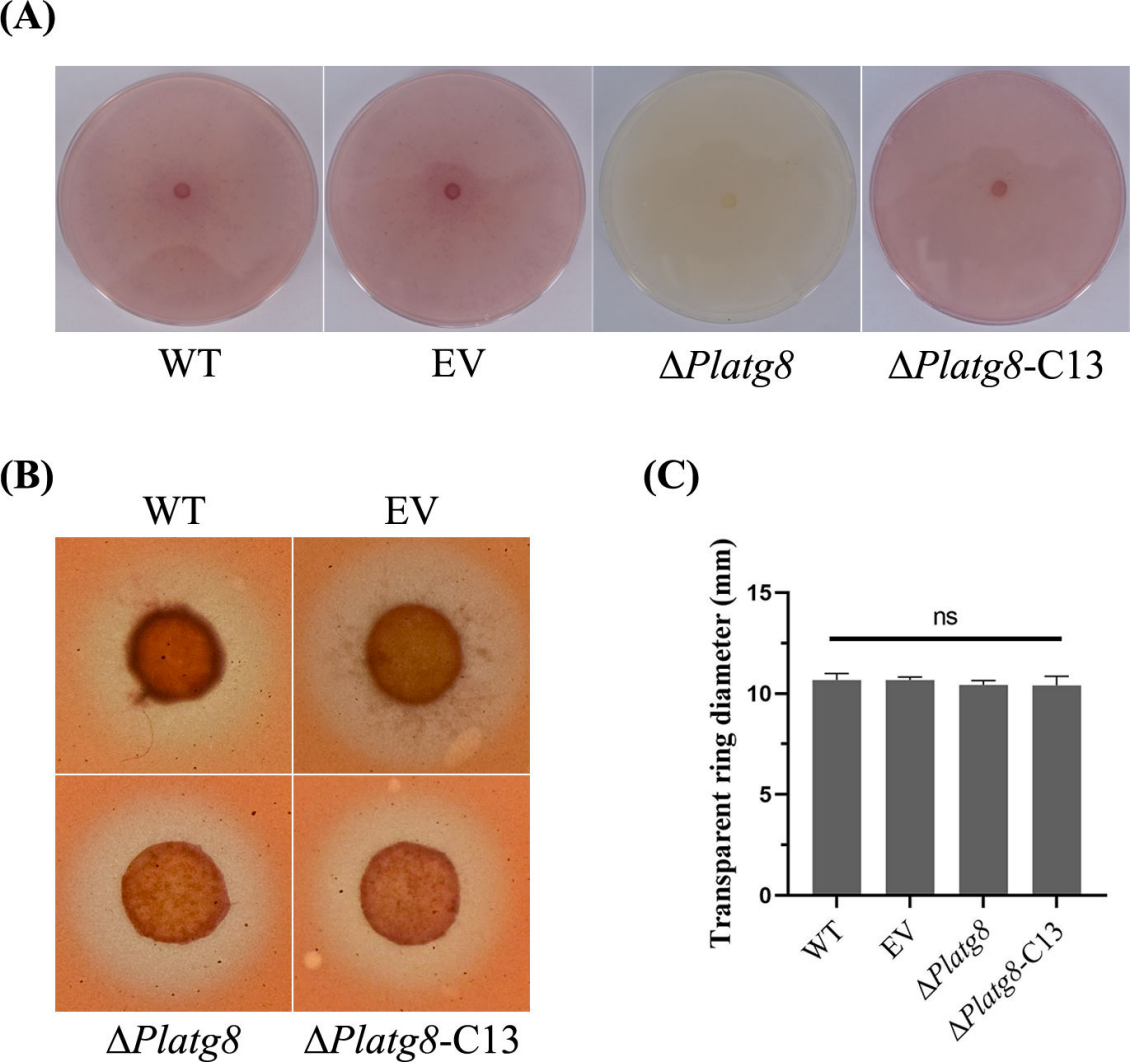
PlAtg8 is required for laccase activity but not for extracellular oxidase. (**A**) Laccase activity analysis of WT, EV, Δ*Platg8*, and Δ*Platg8*-C13 strains. The strains were cultured on Lima bean agar medium with ABTS for 15 days. (**B**) Extracellular oxidase activity analysis of WT, EV, Δ*Platg8*, and Δ*Platg8*-C13 strains. The strains were cultured on V8 medium with CR for 24 h. (**C**) Transparent ring diameter of WT, EV, Δ*Platg8*, and Δ*Platg8*-C13.

Laccase is a copper-containing polyphenol oxidase and is widely found in plants, fungi, and oomycetes (25, 26). Laccase is involved in pigment synthesis, growth and development, and pathogenicity of plant pathogens. To further understand the reason for decreased pathogenicity, the strains of WT, EV, Δ*Platg8,* and Δ*Platg8*-C13 were cultured on lima bean medium with ABTS for 15 days. After 15 days, the plate inoculated with WT, EV, and Δ*Platg8*-C13 showed deep purple but the color of the plate inoculated with Δ*Platg8* did not change (Fig. 6A). Moreover, the strains above all were cultured on Plich medium with Congo Red for 24 h. The results showed that a transparent circle was observed around the inoculation sites of WT, EV, Δ*Platg8,* and Δ*Platg8*-C13, and there was no significant difference in the diameter of the transparent circle between the strains (Fig. 6B and C). These results showed that the deficiency of PlAtg8 does not affect the extracellular oxidase activity but reduces laccase activity in *P. litchii*.

### PlAtg8 is required for stress resistance

**Fig 7:**
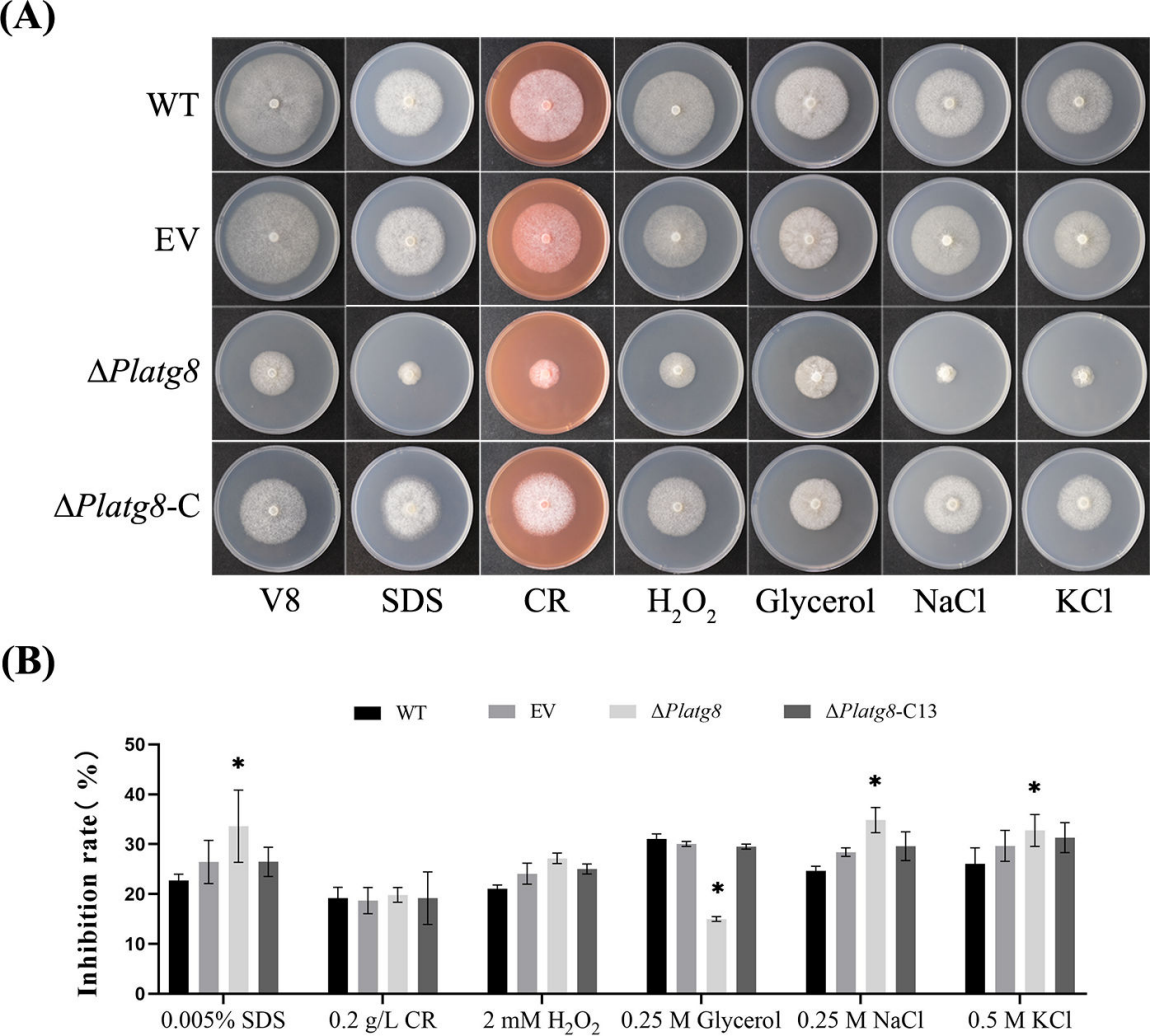
PlAtg8 is required for stress resistance. (**A**) The WT, EV, Δ*Platg8*, and Δ*Platg8*-C13 strains grown on V8 medium or supplemented with 0.005% SDS, 0.2 g/L CR, 2 mM H_2_O_2_, 0.25 mM glycerol, 0.25 M NaCl, and 0.5 M KCl at 25°C for 5 days. (**B**) Growth inhibition rates were calculated for each treatment relative to the growth rate on the V8 medium. Data were analyzed by Duncan’s multiple range test. Asterisks denote significant differences (*P < 0.05). These experiments were repeated three times.

To investigate whether PlAtg8 is involved in osmotic stress, cell wall, and oxidative stress in *P. litchii*, WT, EV, Δ*Platg8,* and Δ*Platg8*-C13 strains were incubated on V8 medium containing different concentrations of SDS, glycerol, CR, sorbitol, NaCl, KCl, and H_2_O_2_, respectively (Fig. 7A). The inhibition rate was calculated after 5 days. The inhibition rate of Δ*Platg8* showed significant differences (*P* < 0.05) compared with WT under all osmotic stresses except sorbitol, suggesting that PlAtg8 influences the resistance of *P. litchii* to osmotic stress (Fig. 7B). Similarly, compared with WT, Δ*Platg8* showed a significant difference (*P* < 0.05) in V8 medium with 0.005% SDS, whereas there was no significant difference in V8 medium with 0.2 g/L CR, suggesting that PlAtg8 has a certain effect on cell wall stress (Fig. 7B). However, Δ*Platg8* had no significant difference (*P* < 0.05) compared to WT in V8 medium with H_2_O_2_, suggesting that PlAtg8 is not involved in oxidative stress in *P. litchii* (Fig. 7B). These results show that PlAtg8 is required for osmotic and cell wall stress but not for oxidative stress.

## DISCUSSION

Autophagy is highly conserved in eukaryotes and is an important pathway for the intracellular degradation of substances (27, 28). Many ATG proteins in filamentous fungi are involved in growth, development, and pathogenicity but ATG proteins in oomycetes are rarely reported. In this study, we identified and analyzed the ATG protein PlAtg8. Through the deletion and complementation of *PlATG8*, we demonstrated that PlAtg8 is required for growth, development, pathogenicity, and stress responses. Specifically, PlAtg8 plays an important role in asexual and sexual reproduction, which is important in the life cycle and disease epidemics of *P. litchii*. Furthermore, localization analysis of PlAtg8 has confirmed that PlAtg8 is indispensable for autophagy, and it could be a helpful marker to study the autophagy of *P. litchii*.

As a core protein of autophagy, Atg8 is well conserved from yeast to humans (29). Atg8 is localized to autophagosomes during autophagy and is degraded after the fusion of the autophagosome with the vesicle (30 – 32). In previous studies, GFP-Atg8 fusion protein can be a helpful marker to monitor autophagy in fungi (16, 17, 33, 34). Through sequence and phylogenetic analysis, we identify the PlAtg8 in *P. litchii*. In addition, the results of MDC staining and the localization of GFP-PlAtg8 showed that PlAtg8 plays an important role in autophagy in *P. litchii*. These results demonstrated that PlAtg8 is conserved among different species and can be a useful marker to study the autophagy of *P. litchii*. However, the mechanism by which autophagosomes mediate vesicular degradation seems different between fungal and oomycete. In *F. graminearum*, GFP fluorescence is detectable in the vacuole when cultured in nitrogen starvation conditions, indicating delivery of autophagic cargo to the vacuole (35). While autophagosome numbers declined over time, GFP fluorescence was not observed in the vacuole when *P. litchii* was cultured in nitrogen starvation conditions. The molecular mechanisms for this phenomenon remain unclear and need further investigation.

Accumulating evidence implicates the role of autophagy in pathogenesis. In *M. oryzae*, deletion of *MoATG8* impaired appressorium function, rendering both mycelia and conidia non-pathogenic (9). Similarly, the deletion of *FgATG8* reduces its virulence because *FgATG8* is implicated in deoxynivalenol (DON) synthesis in *F. graminearum* (10). In addition, the absence of *FgATG8* impaired aerial mycelial growth and reproductive development (36). In this study, deletion of *PlATG8* significantly reduced the pathogenicity of *P. litchii*, impairing growth on leaves and fruits. To further elucidate the basis for decreased pathogenicity in Δ*Platg8*, we analyzed growth and developmental differences between the WT and Δ*Platg8*. Similar to observations in *M. oryzae* and *F. graminearum*, our results showed that the strain Δ*Platg8* exhibited slower mycelial growth, reduced sporangia production, decreased zoospore release, and loss of oospore formation compared to WT in *P. litchii*. The compromised pathogenicity exhibited by Δ*Platg8* may be attributed to these observed developmental deficiencies.

Oomycetes currently rank among the most devastating pathogens of agriculture globally. Despite morphological similarities to fungi, oomycetes are phylogenetically distinct from true fungi. Oomycetes produce diverse spore types including sporangia, zoospores, chlamydospores, and oospores. Among these, sporangia and zoospores are critical in plant-oomycete interactions (23). Sporangial development into either direct germination or zoospore release depends on ambient temperature conditions. Previous studies have shown the G protein signaling pathway is critical for sporangium development and formation (37 – 39). In *P. litchii*, we characterized three G proteins named PlGpa1, PlGpb1, and PlGpg1, which showed elevated expression in the Δ*Platg8* strain. In this study, we found PlAtg8 is required for sporangia production and zoospores release but no interaction was found between the G proteins and PlAtg8 by yeast two-hybrid assays in *P. litchii*. The relationship between G proteins and PlAtg8 remains to be further studied. Before zoospore release, the cytoplasm of sporangia has to cleave (24). In *P. litchii*, PlMapk2 is essential for sporangia cleavage (40). In *P. infestans*, overexpression of *PiGK4* caused impairments in cytoplasmic cleavage and zoospore release. However, the mechanisms underlying sporangia cleavage remain poorly characterized. Here, we demonstrate for the first time an association between autophagy genes and sporangia cleavage. Further investigation is warranted to elucidate the mechanisms linking autophagy to the regulation of this process.

In summary, this paper demonstrates that PlAtg8 is critical for autophagy, development, and pathogenesis in *P. litchii*, highlights differences between oomycetes and fungi, and reveals a novel connection between autophagy and sporangia cleavage.

## Supplementary Material

Reviewer comments

## Data Availability

Data will be made available on request.

## SUPPLEMENTAL MATERIAL

Supplemental material spectrum.03531-23-s0001.docx Fig. S1 and S2; Tables S1 and S2.

OPEN PEER REVIEW reviewer-comments.pdf An accounting of the reviewer comments and feedback.

ASM does not own the copyrights to Supplemental Material that may be linked to, or accessed through, an article. The authors have granted ASM a non-exclusive, world-wide license to publish the Supplemental Material files. Please contact the corresponding author directly for reuse.
