# Supplementary material for: PlAtg8-mediated autophagy regulates vegetative growth, sporangial cleavage, and pathogenesis in Peronophythora litchii
Source: Microbiol Spectr. 2023 Dec 12;12(1):e03531-23. doi: 10.1128/spectrum.03531-23 (PMC10783124; doi:10.1128/spectrum.03531-23)
Supplement: Supplemental material — Fig. S1 and S2; Tables S1 and S2. [file spectrum.03531-23-s0001.docx]

**PlAtg8-mediated autophagy regulates vegetative growth, sporangial cleavage, and pathogenesis in** ***Peronophythora litchii***

Ge Yu^1, 2#^, Wenqiang Li^3#^, Chengdong Yang^1, 2^, Xue Zhang^1, 2^, Manfei Luo^1, 2^, Taixu Chen^1, 2^, Xuejian Wang^1, 2^, Rongbo Wang^3^, Qinghe Chen^1, 2^*

^1^ School of Tropical Agriculture and Forestry, School of Breeding and Multiplication (Sanya Institute of Breeding and Multiplication), Hainan University, Sanya 572025, China.

^2^ Key Laboratory of Green Prevention and Control of Tropical Plant Diseases and Pests, Ministry of Education, Hainan University, Haikou, China;

^3^ Fujian Key Laboratory for Monitoring and Integrated Management of Crop Pests, Institute of Plant Protection, Fujian Academy of Agricultural Sciences, Fuzhou 350003, China.

^#^These authors contributed equally to this work.

*Authors for Correspondence: Dr. Qinghe Chen

E-mail: qhchen@hainanu.edu.cn

**Running title:** PlAtg8-mediated autophagy of *Peronophythora litchii*


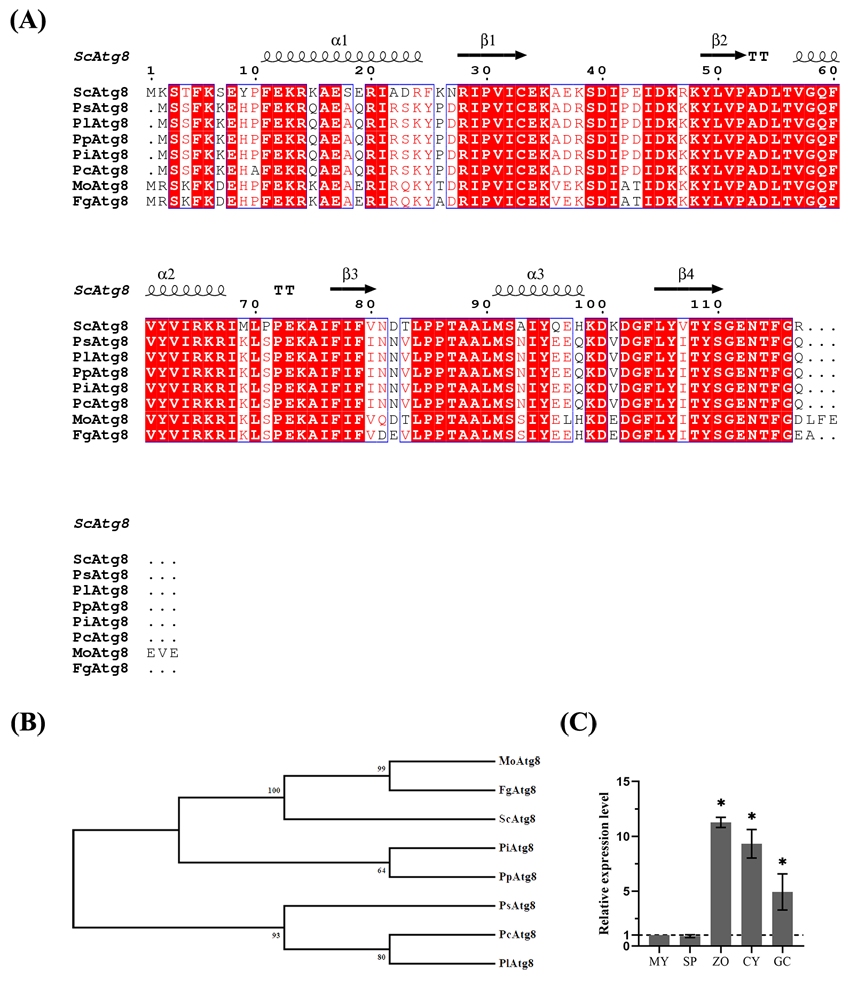


**Fig S1.** Sequence analysis of PlAtg8. (A) Multiple alignment of amino acid sequences of ScAtg8 (*S. cerevisiae*), MoAtg8 (*M. oryzae*), FgAtg8 (*F. graminearum*), PsAtg8 (*P. sojae*), PpAtg8 (*P. parasitica*), PiAtg8 (*P. infestans*), PcAtg8 (*P. capsici*), and PlAtg8 (*P. litchii*). Identical amino acids are shaded in dark red, while amino acids with ≥50 % similarity are highlighted in light red. (B) A phylogenetic tree of Atg8 orthologs from diverse fungal and oomycete species was constructed using the neighbor-joining method in MEGA 11. The sequences included ScAtg8, MoAtg8, FgAtg8, PsAtg8, PpAtg8, PiAtg8, PcAtg8, and PlAtg8. (C) The expression levels of *PlATG8* during the asexual life cycle was analyzed by qRT-PCR. MY: mycelia; SP: sporangia; ZO: zoospore; CY: cyst; GC: germination of cyst. Relative expression levels were calculated using the 2^-ΔΔCT^ method. Asterisks indicate significant difference compared with MY (p < 0.05). These experiments were repeated three times.


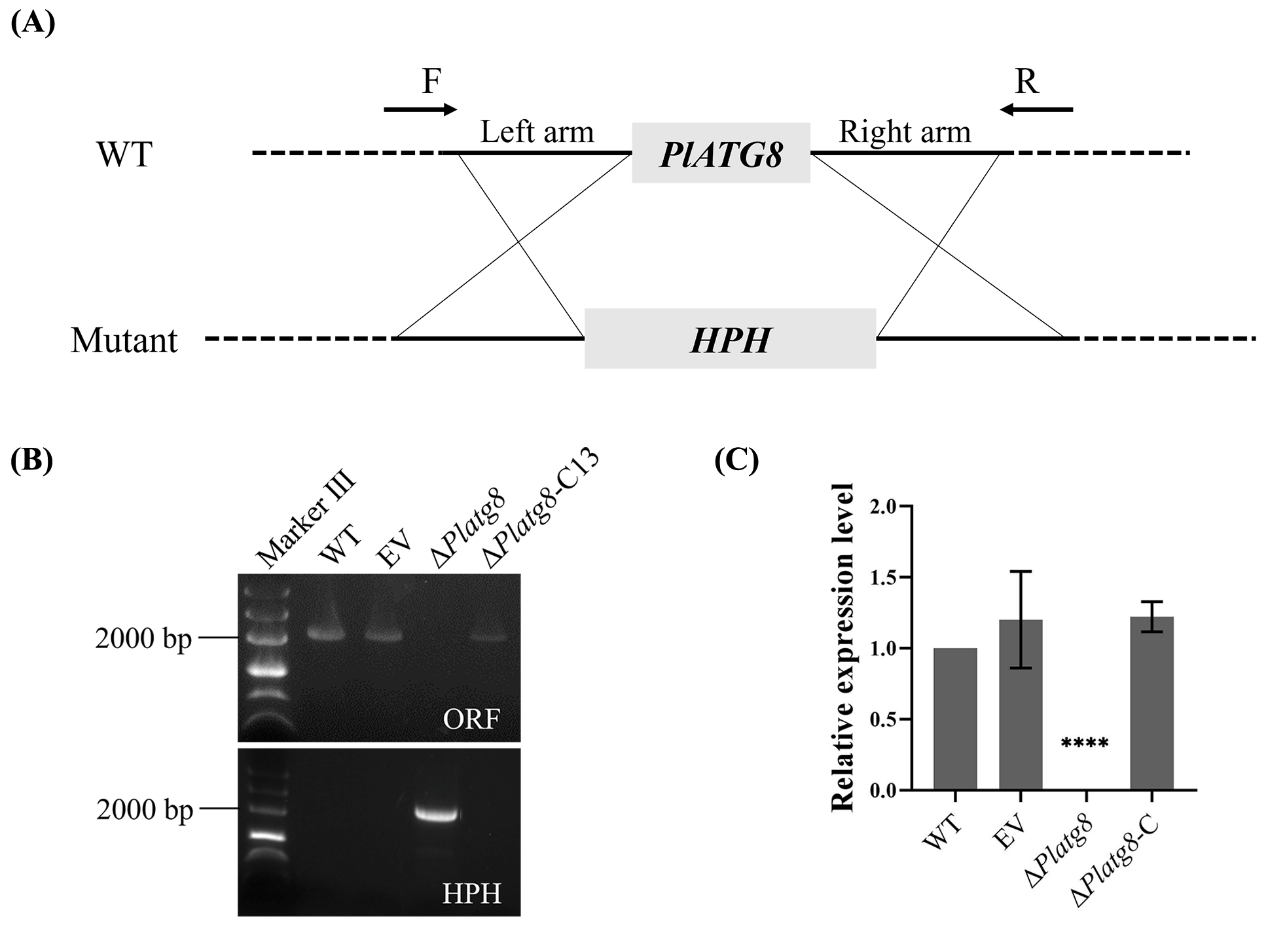


**Fig S2.** Targeted deletion and complement of *PlATG8*. (A) Gene disruption strategy for *PlATG8* in *P. litchii*. The *PlATG8* coding region was replaced with a hygromycin resistance cassette (HPH) by homologous recombination. (B) Identification of the *PlATG8* deletion mutants and complemented strain by PCR amplification. (C) The expression levels of *PlATG8* in WT, EV, Δ*Platg8*, Δ*Platg8*-EV, and Δ*Platg8-C13* was analyzed by qRT-PCR. Relative expression levels were calculated using the 2^-ΔΔCT^ method. Asterisks indicate significant difference compared with WT (****, p < 0.0001). These experiments were repeated three times.

| TABLE S1 Primers used in this study | | |
| --- | --- | --- |
| Primer | Sequence (5' - 3') | Description |
| PlATG8 up 453 F | ACGGCGAATGCCTGAATC | Verifing PlATG8 knockout mutants in *P*. *litchii* |
| PlATG8 dn 372 R | CCAATACTGCCACCTCTTC | Verifing PlATG8 knockout mutants in *P*. *litchii* |
| PlATG8 UP F | CCCCTCGAGGTCGACGGTATCCAATGCACGACACAGAC | Cloning the full length of up arm of PlATG8 |
| PlATG8 UP R | GCTTTTTCATTGTCGCTATGAAAACACTG | Cloning the full length of up arm of PlATG8 |
| Hph F | CATAGCGACAATGAAAAAGCCTGAACTC | Replacing the ORF of PlATG8 |
| Hph R | CACATGCAGCCTACTTATATCAAAGCTTGCC | Replacing the ORF of PlATG8 |
| PlATG8 DN F | ATATAAGTAGGCTGCATGTGCTGTCCGC | Cloning the full length of down arm of PlATG8 |
| PlATG8 DN R | CGGCCGCTCTAGAACTAGTGCAACTGCTGCTGCTGGAC | Cloning the full length of down arm of PlATG8 |
| PlATG8 sgRNA1F | CTAGCGAGGAACTGATGAGTCCGTGAGGACGAAACGAGTAAGCTCGTCTTCCTCTACATCACCTACAG | Constructing pYF515 to target PlATG8 |
| PlATG8 sgRNA1R | AAACCTGTAGGTGATGTAGAGGAAGACGAGCTTACTCGTTTCGTCCTCACGGACTCATCAGTTCCTCG | Constructing pYF515 to target PlATG8 |
| PlATG8 sgRNA2F | CTAGCTTGGTCCTGATGAGTCCGTGAGGACGAAACGAGTAAGCTCGTCGACCAACTGTCAGATCGGCT | Constructing pYF515 to target PlATG8 |
| PlATG8 sgRNA2R | AAACAGCCGATCTGACAGTTGGTCGACGAGCTTACTCGTTTCGTCCTCACGGACTCATCAGGACCAAG | Constructing pYF515 to target PlATG8 |
| M13-F | TGTAAAACGACGGCCAGT | Verifing primers used in knockout vector |
| M13-R | CAGGAAACAGCTATGACC | Verifing primers used in knockout vector |
| qRT-PlATG8 F | CCAGTGATTTGTGAGAAG | qRT-PCR of indicated gene, for measuring gene expression level |
| qRT-PlATG8 R | GTAGGTGATGTAGAGGAA | qRT-PCR of indicated gene, for measuring gene expression level |
| qRT-PlACTIN F | TCACGCTATTGTTCGTCTGG | qRT-PCR of indicated gene, as a reference gene of *P*. *litchii* |
| qRT-PlACTIN R | TCATCTCCTGGTCGAAGTCC | qRT-PCR of indicated gene, as a reference gene of *P*. *litchii* |
| pKNT-GFP F | CCTTCACTCTCACCGACAAGAATTCATGGTGAGCAAGGGCGAG | Cloning the full length of GFP |
| pKNT-GFP R | CTTGTACAGCTCGTCCATG | Cloning the full length of GFP |
| pKNT-PlATG8 F | CATGGACGAGCTGTACAAGATGAGCTCATTCAAGAAG | Cloning the full length of PlATG8 |
| pKNT-PlATG8 R | CACAGCTCGACCTTCGGCAAGCTTCTATTGACCGAAGGTGTTC | Cloning the full length of PlATG8 |

| TABLE S2 Protein sequence used in this study | | |
| --- | --- | --- |
| Primer | Species | Sequence |
| ScAtg8  (YBL078C) | *Saccharomyces cerevisiae* | MKSTFKSEYPFEKRKAESERIADRFKNRIPVICEKAEKSDIPEIDKRKYLVPADLTVGQFVYVIRKRIMLPPEKAIFIFVNDTLPPTAALMSAIYQEHKDKDGFLYVTYSGENTFGR |
| MoAtg8  (MGG_01062) | *Magnaporthe oryzae* | MRSKFKDEHPFEKRKAEAERIRQKYTDRIPVICEKVEKSDIATIDKKKYLVPADLTVGQFVYVIRKRIKLSPEKAIFIFVQDTLPPTAALMSSIYELHKDEDGFLYITYSGENTFGDLFEEVE |
| FgAtg8  (FGRAMPH1_01G20343) | *Fusarium graminearum* | MRSKFKDEHPFEKRKAEAERIRQKYADRIPVICEKVEKSDIATIDKKKYLVPADLTVGQFVYVIRKRIKLSPEKAIFIFVDEVLPPTAALMSSIYEEHKDEDGFLYITYSGENTFGEA |
| PiAtg8  (PITG_03393) | *Phytophthora infestans* | MSSFKKEHPFEKRQAEAQRIRSKYPDRIPVICEKADRSDIPDIDKKKYLVPADLTVGQFVYVIRKRIKLSPEKAIFIFINNVLPPTAALMSNIYEEQKDVDGFLYITYSGENTFGQ |
| PcAtg8  (DVH05_007927) | *Phytophthora capsici* | MSSFKKEHAFEKRQAEAQRIRSKYPDRIPVICEKADRSDIPDIDKKKYLVPADLTVGQFVYVIRKRIKLSPEKAIFIFINNVLPPTAALMSNIYEEQKDVDGFLYITYSGENTFGQ |
| PpAtg8  (PPTG_14822) | *Phytophthora parasitica* | MSSFKKEHPFEKRQAEAQRIRSKYPDRIPVICEKADRSDIPDIDKKKYLVPADLTVGQFVYVIRKRIKLSPEKAIFIFINNVLPPTAALMSNIYEEQKDVDGFLYITYSGENTFGQ |
| PsAtg8  (PHYSODRAFT_353495) | *Phytophthora sojae* | MSSFKKEHPFEKRQAEAQRIRSKYPDRIPVICEKADRSDIPDIDKKKYLVPADLTVGQFVYVIRKRIKLSPEKAIFIFINNVLPPTAALMSNIYEEQKDVDGFLYITYSGENTFGQ |
| PlAtg8 | *Peronophythora litchii* | MSSFKKEHPFEKRQAEAQRIRSKYPDRIPVICEKADRSDIPDIDKKKYLVPADLTVGQFVYVIRKRIKLSPEKAIFIFINNVLPPTAALMSNIYEEQKDVDGFLYITYSGENTFGQ |
