# Supplementary material for: PlAtg8-mediated autophagy regulates vegetative growth, sporangial cleavage, and pathogenesis in Peronophythora litchii
Source: Microbiol Spectr. 2023 Dec 12;12(1):e03531-23. doi: 10.1128/spectrum.03531-23 (PMC10783124; doi:10.1128/spectrum.03531-23)
Supplement: Reviewer comments [file reviewer-comments.pdf]

AMERICAN SOCIETY FOR MICROBIOLOGY | Microbiology Spectrum

PlAtg8-mediated autophagy regulates vegetative growth, sporangial cleavage, and pathogenesis in Peronophythora litchii

Ge Yu, Wenqiang Li, Chengdong Yang, Xue Zhang, Manfei Luo, Taixu Chen, Xuejian Wang, Rongbo Wang, and Qinghe Chen

Corresponding Author(s): Qinghe Chen, Hainan University

Review Timeline:

Submission Date:	September 30, 2023
Editorial Decision:	October 22, 2023
Revision Received:	November 1, 2023
Accepted:	November 13, 2023

Editor: Chengshu Wang

Reviewer(s): The reviewers have opted to remain anonymous.

Transaction Report:

(Note: With the exception of the correction of typographical or spelling errors that could be a source of ambiguity, letters and reports are not edited. The original formatting of letters and referee reports may not be reflected in this compilation.)

DOI: https://doi.org/10.1128/spectrum.03531-23

October 22, 2023

Dr. Qinghe Chen
Hainan University
School of Tropical Agriculture and Forestry
Haikou
China

Re: Spectrum03531-23 (**PlAtg8-mediated autophagy regulates vegetative growth, sporangial cleavage, and pathogenesis in *Peronophythora litchii***)

Dear Dr. Qinghe Chen:

Thank you for submitting your manuscript to Microbiology Spectrum. When submitting the revised version of your paper, please provide (1) point-by-point responses to the issues raised by the reviewers as file type "Response to Reviewers," not in your cover letter, and (2) a PDF file that indicates the changes from the original submission (by highlighting or underlining the changes) as file type "Marked Up Manuscript - For Review Only". Please use this link to submit your revised manuscript - we strongly recommend that you submit your paper within the next 60 days or reach out to me. Detailed instructions on submitting your revised paper are below.

Link Not Available

Below you will find instructions from the Microbiology Spectrum editorial office and comments generated during the review.

ASM policy requires that data be available to the public upon online posting of the article, so please verify all links to sequence records, if present, and make sure that each number retrieves the full record of the data. If a new accession number is not linked or a link is broken, provide production staff with the correct URL for the record. If the accession numbers for new data are not publicly accessible before the expected online posting of the article, publication of your article may be delayed; please contact the ASM production staff immediately with the expected release date.

The ASM Journals program strives for constant improvement in our submission and publication process. Please tell us how we can improve your experience by taking this quick Author Survey.

Sincerely,

Chengshu Wang

Editor, Microbiology Spectrum

Journals Department
American Society for Microbiology
1752 N St., NW
Washington, DC 20036
E-mail: spectrum@asmusa.org

Reviewer comments:

Reviewer #1 (Comments for the Author):

Yu et al sought to explore the biological roles of Atg8 in plant pathogenic fungus Peronophythora litchi. In this study, PlAtg8, the yeast Atg8 homolog, was disrupted by
CRISPR/Cas9 coupled with PEG-mediated protoplast transformation method. The results revealed the PlAtg8 is required for fungal growth, development, and pathogenicity. These findings expend biological functions of autophagy-related genes in fungal pathogens. This investigation was well-planned and data were accurately collected. The manuscript was well organized. It is recommended to be published in this journal.

Minor concerns:
1. Line 31. Change "and" into "coupled with".
2. Line 33-34. Delete this sentence which makes no sense.
3. Line 26-42. Please use the correct tense in showing the results obtained in this study.
4. Line 73-74. There is a mismatch between subject and object.
5. Line 90. Change "except" to "except for".
6. Line 103. Change "enhance" to "contribute to".
7. Line 122. Add "(WT)" behind "wild-type strain.
8. Line 289. Add "disruption of " before "PlAtg8".
9. Line 290. Change "are" to "were".
10. Line 294-295. How does PlAtg8 affect the number of sporangia? So, it is wrong to show the gene functions in this manner. Please correct the similar expression modes through the manuscript.
11. Line 296-297. It needs a literature citation here.

Staff Comments:

Preparing Revision Guidelines
To submit your modified manuscript, log onto the eJP submission site at https://spectrum.msubmit.net/cgi-bin/main.plex. Go to Author Tasks and click the appropriate manuscript title to begin the revision process. The information that you entered when you first submitted the paper will be displayed. Please update the information as necessary. Here are a few examples of required updates that authors must address:

• Point-by-point responses to the issues raised by the reviewers in a file named "Response to Reviewers," NOT IN YOUR COVER LETTER.
• Upload a compare copy of the manuscript (without figures) as a "Marked-Up Manuscript" file.
• Each figure must be uploaded as a separate file, and any multipanel figures must be assembled into one file.
• Manuscript: A .DOC version of the revised manuscript
• Figures: Editable, high-resolution, individual figure files are required at revision, TIFF or EPS files are preferred

For complete guidelines on revision requirements, please see the journal Submission and Review Process requirements at https://journals.asm.org/journal/Spectrum/submission-review-process. **Submissions of a paper that does not conform to Microbiology Spectrum guidelines will delay acceptance of your manuscript.** "

Please return the manuscript within 60 days; if you cannot complete the modification within this time period, please contact me. If you do not wish to modify the manuscript and prefer to submit it to another journal, please notify me of your decision immediately so that the manuscript may be formally withdrawn from consideration by Microbiology Spectrum.

If your manuscript is accepted for publication, you will be contacted separately about payment when the proofs are issued; please follow the instructions in that e-mail. Arrangements for payment must be made before your article is published. For a complete list of **Publication Fees**, including supplemental material costs, please visit ourwebsite.

Corresponding authors may join or renew ASM membership to obtain discounts on publication fees. Need to upgrade your membership level? Please contact Customer Service at Service@asmusa.org.

Thank you for submitting your paper to Microbiology Spectrum.

Dear Editor and Reviewers:

Thank you very much for your comments concerning our manuscript entitled **"PlAtg8-mediated autophagy regulates vegetative growth, sporangial cleavage, and pathogenesis in *Peronophythora litchii*"** (Spectrum03531-23). These comments are all valuable and very helpful for revising and improving our manuscript, as well as the important guiding significance to our research. We have studied comments carefully and have made correction in a point-by-point fashion which we hope meet with approval. The main corrections in the manuscript and the responds to the editor and reviewers' comments are as following:

Responds to the reviewers' comments:

Reviewer: 1

Comment #1: Line 31. Change "and" into "coupled with".

Response: Thanks for the suggestion. We have changed it in the revised manuscript.

Comment #2: Line 33-34. Delete this sentence which makes no sense.

Response: Many thanks for the suggestion. We have deleted the sentence in the revised manuscript.

Comment #3: Line 26-42. Please use the correct tense in showing the results obtained in this study.

Response: Many thanks for this helpful suggestion. We have fixed the error in the revised manuscript.

Comment #4: Line 73-74. There is a mismatch between subject and object.

Response: We have fixed the error in the revised manuscript.

Comment #5: Line 90. Change "except" to "except for".

Response: We have fixed the error in the revised manuscript.

Comment #6: Line 103. Change "enhance" to "contribute to".

Response: Thanks for the suggestion. We have changed enhance to contribute to in the revised manuscript.

Comment #7: Line 122. Add "(WT)" behind "wild-type strain.

Response: Thanks for the suggestion. We have added it in the revised manuscript.

Comment #8: Line 289. Add "disruption of " before "PlAtg8".

Response: Thanks for the suggestion. We have added it in the revised manuscript.

Comment #9: Line 290. Change "are" to "were".

Response: Thanks for the suggestion. We have changed are to were in the revised manuscript.

Comment #10: Line 294-295. How does PlAtg8 affect the number of sporangia? So, it is wrong to show the gene functions in this manner. Please correct the similar expression modes through the manuscript.

Response: Many thanks for this helpful suggestion. We have corrected the error in the revised manuscript.

Comment #11: Line 296-297. It needs a literature citation here.

Response: We have fixed the error in the revised manuscript.

Re: Spectrum03531-23R1 (PlAtg8-mediated autophagy regulates vegetative growth, sporangial cleavage, and pathogenesis in Peronophythora litchii)

Dear Dr. Qinghe Chen:

Your manuscript has been accepted, and I am forwarding it to the ASM production staff for publication. Your paper will first be checked to make sure all elements meet the technical requirements. ASM staff will contact you if anything needs to be revised before copyediting and production can begin. Otherwise, you will be notified when your proofs are ready to be viewed.

Data Availability: ASM policy requires that data be available to the public upon online posting of the article, so please verify all links to sequence records, if present, and make sure that each number retrieves the full record of the data. If a new accession number is not linked or a link is broken, provide production staff with the correct URL for the record. If the accession numbers for new data are not publicly accessible before the expected online posting of the article, publication may be delayed; please contact ASM production staff immediately with the expected release date.

Publication Fees: For information on publication fees and which article types have charges, please visit ourwebsite. We have partnered with Copyright Clearance Center (CCC) to collect author charges. If fees apply to your paper, you will receive a message from no-reply@copyright.com with further instructions. For questions related to paying charges through RightsLink, please contact CCC at ASM_Support@copyright.com or toll free at +1-877-622-5543. CCC makes every attempt to respond to all emails within 24 hours.

ASM Membership: Corresponding authors may join or renew ASM membership to obtain discounts on publication fees. Need to upgrade your membership level? Please contact Customer Service at Service@asmusa.org.

PubMed Central: ASM deposits all Spectrum articles in PubMed Central and international PubMed Central-like repositories immediately after publication. Thus, your article is automatically in compliance with the NIH access mandate. If your work was supported by a funding agency that has public access requirements like those of the NIH (e.g., the Wellcome Trust), you may post your article in a similar public access site, but we ask that you specify that the release date be no earlier than the date of publication on the Spectrum website.

Embargo Policy: A press release may be issued as soon as the manuscript is posted on theSpectrum Latest Articles webpage. The corresponding author will receive an email with the subject line "ASM Journals Author Services Notification" when the article is available online.

The ASM Journals program strives for constant improvement in our submission and publication process. Please tell us how we can improve your experience by taking this quick Author Survey.

Thank you for submitting your paper to Spectrum.

Sincerely,
Chengshu Wang
Editor
Microbiology Spectrum

Reviewer #1 (Comments for the Author):

All concerns have been addressed, and the manuscript is acceptable for publication.
